# The effect of melatonin treatment on postural stability, muscle strength, and quality of life and sleep in postmenopausal women: a randomized controlled trial

**DOI:** 10.1186/s12937-015-0093-1

**Published:** 2015-09-30

**Authors:** Anne Kristine Amstrup, Tanja Sikjaer, Leif Mosekilde, Lars Rejnmark

**Affiliations:** Department of Endocrinology and Internal Medicine (MEA), Aarhus University Hospital, Tage-Hansens Gade 2 DK-Aarhus C, 8000 Aarhus, Denmark

**Keywords:** Osteopenia, Women, Balance, Muscle function, Melatonin

## Abstract

**Background:**

Melatonin is often used as a sleeping aid in elderly adults. As previous studies suggest a protective role of melatonin against osteoporosis, it is important to document its safety. Treatment should not cause any hangover effect that could potentially lead to falls and fractures. We therefore aimed to evaluate the effect of melatonin on balance- and muscle function.

**Methods and patients:**

In a double-blind placebo-controlled study, we randomized 81 postmenopausal women with osteopenia to receive 1 or 3 mg melatonin, or placebo nightly for 12 months. Postural balance as well as muscle function was measured. In addition, we assessed quality of life and sleep at baseline and after 12 months treatment.

**Results:**

Compared to placebo, one-year treatment with melatonin did not affect postural balance or risk of falls. Furthermore, no significant changes between groups were observed in muscle strength in neither upper- nor lower extremities. Treatment did not affect quality of life or sleep. However, in the subgroup of women with sleep disturbances at baseline, a trend towards an improved sleep quality was seen (*p* = 0.08).

**Conclusion:**

Treatment with melatonin is safe in postmenopausal women with osteopenia. There is no hangover effect affecting balance- and muscle function following the intake of melatonin. In women with a good quality of sleep, melatonin has no effect, however in poor quality of sleep, small doses of melatonin trended towards improving the quality.

**Trial registration:**

(# NCT01690000)

## Introduction

Melatonin in known for its regulation of circadian rhythm. It is produced in the pineal gland and is stimulated by darkness while inhibited by light [1]. In humans, the production decreases by age [2], and treatment with melatonin may be used as a sleeping aid in elderly people.

In some European countries and the United States, small doses of melatonin are considered a dietary supplement. In other European countries, including Denmark, melatonin is still a prescription drug against primary insomnia recommended to patients >55 years of age in a dose of 2 mg taken at bedtime. Despite an unknown prevalence of the usage of melatonin, there is an increasing interest in melatonin in addition to its effects as a sleeping aid. Melatonin has also shown to be of importance for a number of other physiological functions as experimental and clinical studies have shown a stimulation of the immune system [3], protection against aging [4], cancer [5] and hypertension [6]. Moreover, melatonin may be of importance to bone, as it has been shown to upregulate osteoprotegerin (OPG) and suppress receptor activator of NK-κB ligand (RANKL) [7, 8]. Furthermore, studies have demonstrated an increased stimulation of the osteoblastic cell lineage with suppression of peroxisome proliferator-activated receptor gamma (PPARγ) and enhanced expression of bone morphogenic proteins (BMPs) 2 and 4 [9, 10]. In response to treatment with melatonin, a recent randomized, placebo-controlled study by our group demonstrated an increase in arealBMD in the femoral neck as assessed by DXA, and in volumetricBMD in the lunbar spine as assessed by quantitative computed tomography (QCT) in postmenopausal women with osteopenia (low bone mass with a T-score between -1 and -2.5 [11, 12]).

In general, melatonin is considered a safe treatment option in the elderly. However, if melatonin is going to be used for long term prevention and treatment of osteoporosis (low bone mass with a T-score < −2.5 [11]), it is of specific importance to document its safety. Treatment should not cause any hangover effect (e.g. dizziness or drowsiness) resulting in decreased postural balance and muscle strength that could potentially lead to falls and fractures.

Therefore, as a part of our double-blind randomized placebo-controlled trial on the effect of melatonin in postmenopausal women with osteopenia [12], we evaluated the effects of one-year treatment with melatonin in a daily dose of 1 or 3 mg administered at bedtime on postural balance and muscle function. Furthermore, quality of life and sleep were assessed to see if any effects are present in otherwise healthy women without known sleep disturbances.

## Methods

### Study population

We performed a double-blinded randomized placebo-controlled study. Inclusion criteria were postmenopausal women diagnosed with osteopenia (T-score −1 to–2.5 in either hip or spine). There were no requirements to quality of sleep prior to entering the study. We invited 202 women, and 150 responded positively, hereof 65 were excluded due to exclusions criteria or declining participation after further information. Ultimately, 85 women accepted the invitation, and the first 81 (aged 56–73 years) to accept were randomised to treatment or placebo [12]. The participants were recruited by letter from our outpatient clinic as all women were already diagnosed with osteopenia. The exclusions criteria were, in brief women with known medical conditions affecting the bone. This included p-creatinine >120 μmol/L, P-ionized calcium >1.32 mmol/L, intestinal malabsorption, impaired lever function, smokers and users of drugs with effects on calcium homeostasis. Furthermore, users of antiresorptives, hormone replacement therapy and alcohol abusers (>14 units/wk) were also excluded [12]. The patients were randomized to receiving either 1 or 3 mg of melatonin, or identical placebo daily for 12 months. The tablets were given orally at bedtime. Melatonin as well as identical placebo was manufactured by Skanderborg Pharmacy. As previously mentioned, melatonin is a prescription drug in several European countries recommended in doses of 2 mg. Therefore, to ensure no discomfort among the study subjects, besides the reports from the use of 2 mg, we chose doses close hereto. Furthermore, all participants received a daily supplement of 800 mg calcium and 20 μg vitamin D3. All participants provided informed consent prior to participation in the study. The study was performed in accordance with the Helsinki II declaration.

The study was approved by the local ethic committee of central Denmark (#M2012-252-12), the Danish National Board of Health (EudraCt nr 2011-004670-28), the Danish Data Protecting Agency, and registered by ClinicalTrials.gov (# NCT01690000). The Good Clinical Practice (GCP) unit at Aarhus University Hospital, Denmark monitored the project. All study subjects provided a written consent before entering the study.

### Postural stability

We assessed postural stability by using a stadiometer (Good Balance Platform System™, Metitur Ltd. Finland) to measure body sway. The stadiometer is a triangular platform used to convert shifts in weight to digital data to obtain an assessment of maintenance of balance. The platform reports length (millimetres) and speed (millimetres/second) of the sway in medio-lateral and anterior-posterior direction. The interclass correlation coefficient was 0.87-0.96 [13].

Under four settings, we measured postural stability:

1. Normal standing eyes open (EO). The participant is placed with the feet next to each other with 20 cm apart. The arms are in relaxed position hanging freely at each side and with a fixed gaze on a marked spot in eye level.
2. Normal standing eyes closed (EC). The participant is placed in the same position as the previous exercise but with the eyes closed.
3. Semi-tandem. The heel on the foot on the non-dominant hand side is placed alongside the big toe on the dominant hand’s side. Arms and gaze are in accordance with the first measure.
4. Tandem. The non-dominant hand’s foot is placed in front of the dominant hand’s foot. Eyes and arms as described in measure 1.

The exercises were performed barefoot. The duration of each exercise was 20 second and repeated three times. Data are presented as the velocity moment (VM mm^2^/s), which is calculated as 90 % of the product of the actual distance of movement in the medio-lateral and anterior-posterior direction from the center of pressure per second. We adjusted for the effect of body height, and vertical location of the center of body mass by Scaled Velocity Moment (SVM) = (VM/(height in cm)^2^) × 180^2^. The best measure for each exercise was chosen for further analysis (i.e. the lowest VM).

### Muscle strength

The maximum voluntary isometric muscle strength at the upper and lower extremity was measured with an adjustable dynamometer chair connected to a computer (Good Strength™, Metitur Ltd, Finland) [14]. The device has demonstrated high reliability coefficients (0.88-0.96) for both upper and lower extremities [15]. Recording time of each measure was 5 seconds. Upper extremity strength was assessed by hand grip, and elbow flexion and extension with the elbow in 90 dgr flexion from neutral position. Knee extension and flexion were measured in a 60 dgr and 90 dgr angle from fully extended leg. All measurements were performed while sitting in the chair. The trunk was supported with three belts to minimize transfer of strength from other sites. The excises were performed on the dominant hand’s side and repeated three times with a 30-second break between the recordings. The best performance was chosen for further analysis. Maximum strength was measured in newton (N).

### Quality of life

We used a Danish versions of the Short Form questionnaire 36 version 2 (SF-36) and WHO-Five Well Being Index (WHO-5) to assesses quality of life and well-being. The SF-36 questionnaire consist of 36 questions categorized into 8 subdomains describing physical functioning (PF), role-physical (RP), bodily pain (BP), general health (GH), vitality (VT), social functioning (SF), role-emotional (RE), and mental health (MH). The subdomains are summed up to a mental component score (MCS) and a physical component score (PCS) and can be calculated using norm-based values according to the user’s manual [16].

The Who-5 index consists of five questions, and answers are scored from 0 (worst) to 5 (best). The scores are summed up, multiplied by four, and presented as a total percentage from 0–100. A well-being score below 50 % indicates a depressive affection [17].

Both questionnaires are well used and validated [18].

### Quality of sleep

A Danish version of Pittsburgh Sleep Quality Index (PSQI) [19] was used to assess the quality of sleep. The questionnaire comprises of 21 questions generated into seven components: Subjective sleep quality, sleep latency, sleep duration, habitual sleep efficiency, sleep disturbances, use of sleep medication and daytime dysfunction. The subdomains are summed up to a global score; scoring five or below indicating a good quality of sleep while a score above five is associated with a poor quality of sleep.

### Physical Activity Scale (PAS)

We assessed physical activity by a Danish version of the Physical Activity Scale (PAS) questionnaire in which the participants reported time spend on sports, work, and leisure time on an average weekday [20]. The questionnaire is organized into nine questions regarding activity corresponding to a known metabolic equivalents (MET, 1 kcl/kg/h) level ranging from low activity to high intensity activity: sleep (0.9 MET), TV-viewing/reading (1.0 MET), sitting/working (1.5 METs), standing up (2.0 METs), light work (3.0 METs), light to moderate activity (4.0 METs) moderate activity (5.0 METs), moderate to high activity (6.0 METs) and high intensity activity (7 METs) [20]. Each question is imaged and described by examples of the specific activity. A total MET-score was calculated as minutes and hours spent on each activity multiplied by the assigned MET value, and adding the nine MET activity levels together.

### General questionnaire

Our participants filled out a questionnaire regarding life style including question about use of medication, previous fractures, and daily intake of dietary calcium.

### Blood pressure

In calm settings, and with the study subject in a resting state, we measured blood pressure and heart rate. For the purpose, we used an automatically inflated brachial cuff connected to an oscillometric reading system measuring heart rate and blood pressure.

### Clinical visits

Questionnaires, and balance- and muscle function tests were performed at baseline and after 12 months treatment. At baseline and after 3, 6, 9, and 12 months of treatment the women came for a clinical visit answering question about falls, fractures and adverse advents, and to have their blood pressure measured. Balance- and muscle function were assessed during the morning or afternoon while all clinical visits were carried out in the morning. The same investigator conducted all visits and supervised all exercises to ensure a uniform instruction to all study subjects.

### Statistics

As this is a part of our randomized placebo-controlled study on women with osteopenia, sample size was calculated on the basis of our main outcome with a predicted 1.5 % change in BMD between the melatonin and placebo group. This ultimately led to a total of 72 study subjects (80 % statistical power and 5 % significance). In the present study, to show a 10 % change in knee extension 60° in healthy women (mean force 393 N, SD ±53 N) [21] a total number of 30 study subjects in each groups was required (80 % statistical power, 5 % significance).

We analysed data using with the intention to treat approach as results from all randomized subjects with a follow-up value were included according to the treatment allocation. Differences between groups were tested by the Student’s t test or Wilcoxon Rank-Sum test, as appropriate. Serial changes of blood pressure values were analysed by variance for repeated measures (ANOVA). Interaction between group and time (gr. vs. time) were studied in order to determine whether treatment affected changes blood pressure differentially during the study. Area under the curve (AUC) was calculated in order to assess differences between groups in average blood pressure.

We used Pearson Chi-square or Fischer’s Exact Test for categorical variable. Correlations between variables were tested by bivariate correlation analyses (r). Data are presented as mean with 95 % confidence interval (CI) or median with 25–75 % interquartile range (IQR). The significant level was *p* < 0.05. We used IBM SPSS Statistics 21 to perform all calculations.

## Results

Forty-one women were allocated to placebo, while 40 were allocated to either 1 or 3 mg melatonin. Descriptive data are shown in Table 1. In general, randomization was well balanced with no significant differences between groups.

**Table 1:** Descriptive data at baseline. Mean (±standard deviation) or median (25–75 % interquartile range)

	Placebo *N* = 41	Melatonin *N* = 40	*P*-value
Age, years	62.9 (±4.7)	62.4 (±3.5)	0.46
BMI (kg/m^2^)	24.9 (21.4;26.6)	23.6 (21.3;27.5)	0.38
Weight (kg)	66.6 (60.0;76.0)	65.0 (58.3;75.5)	0.58
Systolic blood pressure (mmHg)	143 (130;149)	138 (128;144)	0.15
Diastolic blood pressure (mmHg)	83 (77;92)	85 (76;91)	0.76
Heart rate	68 (64;75)	70 (64;76)	0.94
Dietary calcium (mg/d)	950 (800;1125)	975 (800;1100)	0.66
Previous fracture after the age of 55 years, (N %)	6 (15 %)	8 (20 %)	0.57
Prescribed medication, N (%)	21 (51 %)	20 (50 %)	0.99
Simvstatin, N (%)	5 (12 %)	3 (8 %)	0.71
Antidepressives, N (%)	2 (5 %)	2 (5 %)	0.99
Benodiazepines, N (%)	1 (2 %)	0	0.99
Antihypertensives, N (%)	6 (14 %)	4 (10 %)	0.74
Levothyroxine, N (%)	1 (2 %)	1 (2 %)	0.99
Pittsburgh Sleep Quality Index > 5	20 (49 %)	9 (23 %)	**0.02**

During our one-year trial including 81 women, we recorded in total 36 adverse events (AE) and 12 serious adverse events (SAE). The events occurred at equal frequency in both groups. In all, we recorded two falls in each group. Two of the falls (one in each group) were caused by snow covering irregularities in the pavement. The other two falls were caused by sightseeing as the women overlooked irregularities in the pavement. Seventy-two women completed the study. Of the nine women dropping out during the trial, five were in the placebo group while four were in the melatonin group [12]. The treatment was well-tolerated and only 16 (40 %) of the women in the melatonin group guessed their randomization compared with 29 (74 %) in the placebo group. The few adverse events and the good-tolerance was the reason why they believed to receive placebo in any of the experimental settings.

At baseline the placebo group had a reduced postural stability by normal standing EO compared to the melatonin group (Table 2). However, 12 months of treatment with melatonin did not affect postural stability compared with placebo.

**Table 2:** Postural balance (scaled velocity moment [m^2^/s]). Baseline and percentages changes after 12 months of treatment with melatonin or placebo (median with 25–75 % interquartile range

	Baseline (m^2^/s)	*P*-value	Changes after 12 months (%)	*P*-value*
Normal standing, eyes open		**0.04**		0.11
Placebo	4.1 (2.9;6.4)		−10.98 (−30.39;19.00)	
Melatonin	3.3 (2.7;4.1)		6.30 (−22.83;43.43)	
Normal standing, eyes closed		0.31		0.29
Placebo	5.7 (3.6;8.5)		−7.79 (−39.72;33.31)	
Melatonin	4.7 (3.4; 6.7)		4.06 (−24.17;50.00)	
Semi tandem		0.58		0.67
Placebo	20.4 (15.6;33.1)		−4.97 (−33.98;29.42)	
Melatonin	20.5 (16.4; 28.7)		−15.60 (−26.79;23.33)	
Tandem		0.48		0.55
Placebo	48.7 (34.1;63.3)		−4.00 (−38.45;48.70)	
Melatonin	43.3 (29.0; 73.3)		2.10 (−26.27;46.77)	

Significance is shown in bold

*Percentages change between groups

Table 3 shows the results of the muscle strength measures. No significant differences between groups were present at baseline. One-year treatment with melatonin did not affect upper or lower extremity performance in any of the groups.

**Table 3:** Maximum voluntary muscle strength (Newton [N]). Baseline data and percentage change after 12 months of treatment according to group allocation. Median with interquartile range (25–75 % percentiles)

Muscle group		*P*-value		*P*-value*
	Baseline (Newton)		Change after 12 months (%)	
Hand grip		0.79		0.58
Placebo	287 (257;311)		−0.18 (−7.02;6.78)	
Melatonin	281 (244;333)		−0.75 (−10.40;7.59)	
Elbow extension		0.23		0.74
Placebo	116 (98;138)		6.21 (1.23;12.85)	
Melatonin	111 (92;121)		5.20 (−3.51;15.28)	
Elbow flexion		0.18		0.37
Placebo	178 (156;192)		1.95 (−6.77;10.75)	
Melatonin	165 (152;185)		−1.45 (−9.12;6.91)	
Knee extension 90°		0.67		0.99
Placebo	309 (278;377)		0.33 (−2.68;5.44)	
Melatonin	312 (275;357)		0.55 (−6.66;11.26)	
Knee flexion 90°		0.40		0.93
Placebo	160 (137;177)		0.26 (−7.13;16.89)	
Melatonin	153 (121;177)		0.00 (−6.09;13.79)	
Knee extension 60°		0.26		0.33
Placebo	384 (326;433)		0.76 (−4.08;4.16)	
Melatonin	370 (313;410)		1.73 (−5.94;10.49)	
Knee flexion 60°		0.44		0.54
Placebo	169 (139;203)		4.31 (−9.61;22.58)	
Melatonin	163 (138;182)		0.99 (−2.53;9.66)	

*Significant changes between groups

Stratifying for doses of melatonin did not change the results in either balance- nor muscle function tests (data not shown).

Table 4 shows the results of the questionnaire data. According to quality of sleep index (PSQI), both groups had a good quality of sleep at baseline (5 in placebo group vs. 4.5 in melatonin group). No significant changes between groups were found in response to treatment. Restricting analyses to study subjects with a score above normal range, i.e. associated with a poor quality sleep/insomnia, this was reported more frequently by women in the placebo group compared with women in the melatonin group (49 vs. 23 %, respectively, *p* = 0.02) (Table 1). In response to treatment, the women in the melatonin group rated with insomnia reduced their score by −37.5 % (IQR: −64.5;−15.5) compared with −13.3 % (IQR: −32.1;9.4) in the placebo group (*p* = 0.08 for between group changes).

**Table 4:** Quality of life, sleep quality, and physical activity. Baseline data and changes (%) in response to 12 months of treatment with melatonin or placebo. Median scores with 25–75 % interquartile range

	Baseline	*P*-value	Changes after 12 months (%)	*P*-value*
Physical component score		0.69		0.16
Placebo	54.9 (48.2;56.9)		1.80 (−3.79;7.11)	
Melatonin	54.7 (50.9;57.4)		−0.30 (−4.00;4.83)	
Mental component score		0.12		0.94
Placebo	56.6 (51.8;59.0)		−0.78 (−5.88;4.47)	
Melatonin	58.0 (55.6;59.5)		−0.34 (−6.13;4.12)	
WHO-5 Well-being Index		0.63		0.27
Placebo	80.0 (72.0;84.0)		0.00 (−9.52;5.00)	
Melatonin	80.0 (73.0;84.0)		0.00 (−5.00;5.56)	
Pittsburgh Sleep Quality Index		0.36		0.31
Placebo	5.0 (2.0;8.5)		0.00 (−32.14;38.33)	
Melatonin	4.5 (3.0;5.0)		0.00 (−41.11;28.79)	
Pittsburgh Sleep Quality Index > 5		0.47		0.08
Placebo, *N* = 20	8.5 (6.0;9.8)		−13.3 (−32.1;9.4)	
Meltonin, *N* = 9	8.0 (6.0;8.5)		−37.5 (−64.5;-15.5)	
Physical activity scale^a^		0.17		0.43
Placebo	43.8 (37.4;48.7)		−0.16 (−8.46;8.29)	
Melatonin	46.0 (40.9;51.3)		−2.27 (−13.84;10.07)	

*Percentage change between groups

^a^Metabolic equivalent (MET) score for 24 hours

In the subgroup of women with insomnia, 58 % of the women guessed their randomization i.e. 86 % in the placebo group and 14 % in the melatonin group (*p* = 0.01). The percentage change in PSQI-score did not differ in women believing to receive melatonin compared to those believing to receive placebo (data not shown).

There were no differences in quality of life between groups in any of the subdomains (data not shown), MCS or PCS at baseline or at follow up. In all the subdomains as well as MCS and PCS, both groups scored within the normal range compared to norm-based values. Who-5 index did not change over time between the groups (Table 4). A significant inverse correlation was present between changes in PCS and changes in PSQI (*r* = −0.36, *p* < 0.01) meaning that the better PCS, the better quality of sleep. No correlation was present between PSQI and other physical parameters such as PAS, muscle and balance function (data not shown). No changes between groups were observed when assessing physical activity (PAS).

**Fig. 1:**
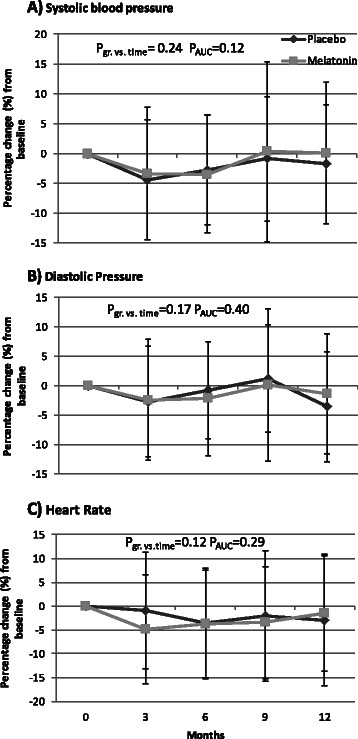
Effects of treatment with placebo or melatonin on blood pressure and heart rate. Mean ± SD

Heart rate and blood pressure did not differ between groups at baseline. Compared with placebo, melatonin caused a non-significantly decrease in systolic (5 mmHg, *p* = 0.12) and diastolic (2 mmHg, *p* = 0.40) blood pressure (Fig. 1). Heart rate did not change in response to treatment (data not shown).

## Discussion

In the present study we investigated the effects of one-year treatment with melatonin on balance- and muscle function as well as physical and mental well-being in postmenopausal women with osteopenia. Our study did not raise safety concerns regarding reduced postural balance- or muscle function. Furthermore, in postmenopausal women with a normal sleep quality, treatment with low doses of melatonin did not affect quality of life or sleep. In the subgroup of people with insomnia there was, however, a borderline significant improvement in quality of sleep in the melatonin group compared to placebo.

To the best of our knowledge this is the first study to evaluate the effect of longer term treatment with small doses of melatonin on balance- and muscle function in postmenopausal women. Melatonin is commonly used as a sleep aid in the elderly. As melatonin increased bone mineral density [12] through previously mentioned possible mechanisms, it may also in the future play a role in the prevention and treatment of osteoporosis. It is therefore of importance to rule out any long term adverse effects that may increase the risk of falls and fractures. The manufacture of 2 mg melatonin describes the side effects in terms of hangover (dizziness and sleepiness during the day) as events occurring in 0.1–1 % of the patients [22]. The half-life of melatonin is dependent on the composition of the drug varying from app. 30 min to four hours, and may be affected by e.g. fasting state [22–25]. Administered just before bedtime, melatonin should be eliminated in the morning. However, as described by the manufactures of 2 mg melatonin, hangover during the day may still occur, which is probably caused by intersubject viability.

In previous studies, discrepant results have been reported on the effects of melatonin on balance performance. In one study [26], an oral intake of 10 mg of melatonin was found to impair postural stability, whereas Otmani et al. [27] reported no detrimental effects of 2 mg short term treatment of melatonin on postural balance. Furthermore, the hangover effect of melatonin has previously been examined in relation to muscle strength in young adults showing no alteration in response to a single dose of 5 mg melatonin [28, 29]. In the present study we evaluated the effect of melatonin in response to one-year treatment. A potential adaption to side effects causing no changes in balance and muscle function cannot be completely ruled out. However, contradicting this postulate, we reported an equal frequency of falls in the groups throughout the trial. Furthermore, to avoid learning effect to the exercises, it is of rational not to repeat the measurements until one-year of treatment.

In a previous study by Kotlarczyk et al. [30], the authors investigated the effect of 3 mg melatonin/d for 6 months in 18 healthy perimenopausal women. In line with our results, no effect of melatonin was seen in relation to the average quality of sleep. This is most likely explained by the fact that both studies comprised of subjects without known sleep disturbances, and the results are further supporting the findings by other investigators [31]. It is, however, well known that melatonin has a positive effect on sleep in patients with insomnia [32, 33]. Similar to these results we did find a trend towards a positive effect of melatonin on quality of sleep in the subgroup of people with pre-exiting poor quality of sleep. The lack of statistical significance is most likely explained by a too low statistical power.

Concerning the self-rated quality of life questionnaires, we conclude that melatonin does not affect the outcome measures negatively. Our results are in accordance with Kotlarczyk et al. [30] who demonstrated a safe use of melatonin with no significant changes in domains, except physical, as assessed by Menopause-Specific quality of life (MENQOL) questionnaire. The physical domain increased in the study in response to melatonin. Our results showed a significantly inverse correlation between the physical component score (PCS) and quality of sleep indicating that the better the physical score, the better the sleep. Although an effect of melatonin in relation to quality of life has previously been shown in patients with known sleep disturbances [33] our study consisted of healthy women with a good quality of life and an increase was not expected.

There have previously been demonstrated a reduction in nocturnal systolic and diastolic blood pressure after nightly therapeutic doses of 2–3 mg melatonin [6, 34, 35]. However, it line with previous results [30], we did not observe significant effects of melatonin on blood pressure. This may be explained by the fact that we did not measure blood pressure during night after ingestion of melatonin.

There are several strengths to the study including its design as a double-blind randomized placebo- controlled trial. Furthermore, as to evaluate the effect of melatonin, the study subjects were in the relevant age group. There are however, also limitations to the study, as the women did not suffer from sleep disturbances prior to entering the study. However, a positive effect was not anticipated as our primary goal was to establish the safety aspects of longer term treatment with melatonin.

In conclusion, melatonin in a daily dose of 1 or 3 mg is safe to use in postmenopausal women with osteopenia. There is no long term hangover effect causing a reduction in balance- and muscle function or quality of life. In women with poor quality of sleep, small doses of melatonin trended towards improving quality of sleep.
